# Supplementary figures and images for: Analyses and Insights into Genetic Reassortment and Natural Selection as Key Drivers of Piscine orthoreovirus Evolution
Source: Viruses. 2024 Apr 2;16(4):556. doi: 10.3390/v16040556 (PMC11053957; doi:10.3390/v16040556)

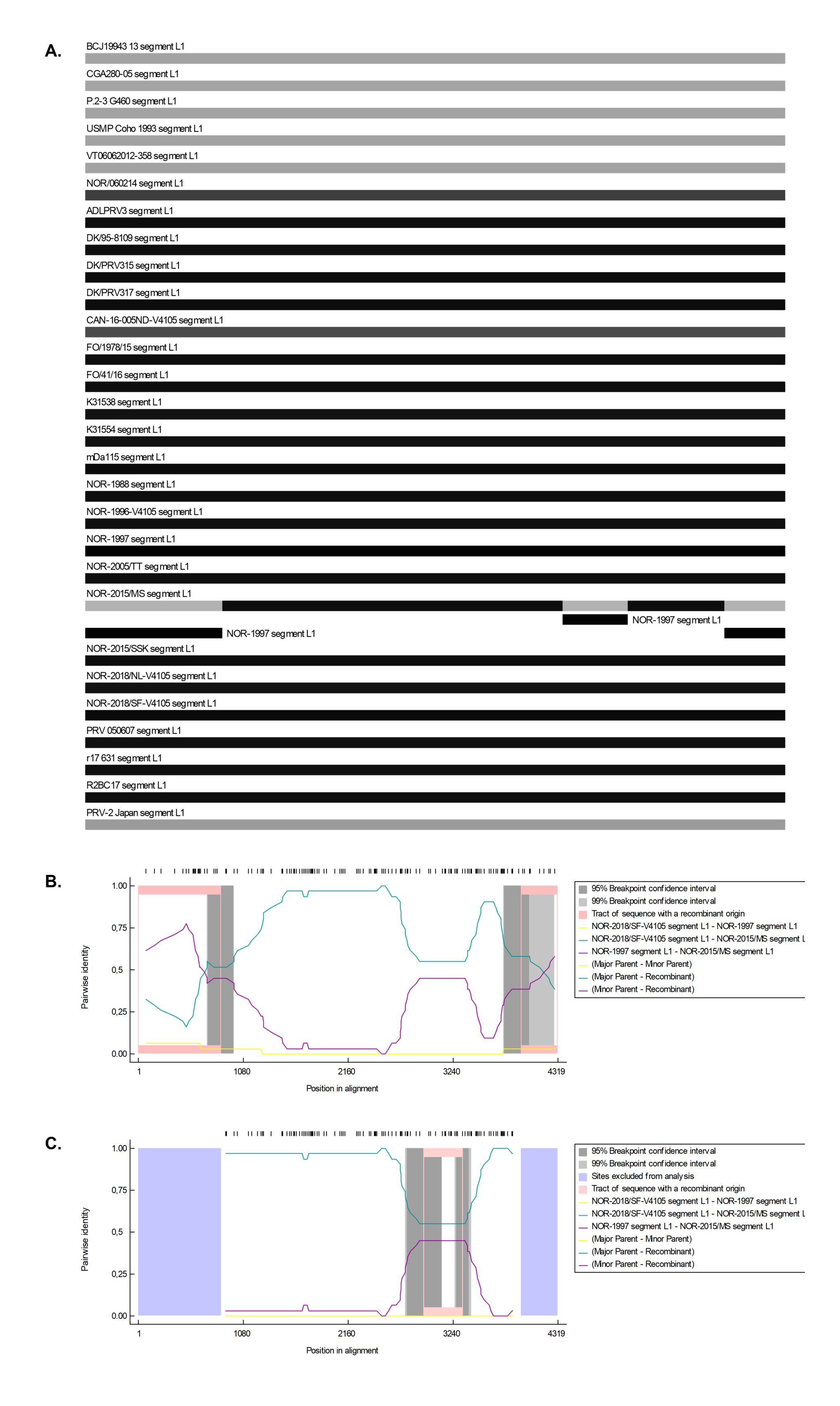

Supplement: Supplementary file 1 [file viruses-16-00556-s001.zip › FigureS1_SupplementaryFigureS1.tif]
